# Clear Cell Carcinoma: A Rare Variant of Cholangiocarcinoma Case Report and Systematic Review

**DOI:** 10.1155/crgm/1716741

**Published:** 2025-06-28

**Authors:** Salah Abdel Jalil, Majd M. AlBarakat, Ghazi Abu Afifeh, Rana B. Altawalbeh, Ala Abdel-Jalil

**Affiliations:** ^1^Department of Surgery, Atrium Health, Macon, Georgia, USA; ^2^Faculty of Medicine, Jordan University of Science and Technology, Irbid, Jordan; ^3^MetroHealth Medical Center, Case Western Reserve University, Cleveland, Ohio, USA; ^4^Faculty of Medicine, University of Jordan, Amman, Jordan; ^5^Gastroenterology & Hepatology, MetroHealth Medical Center, Case Western Reserve University, Cleveland, Ohio, USA

**Keywords:** abdominal pain, clear cell carcinoma, hepatectomy, intrahepatic cholangiocarcinoma

## Abstract

**Background:** Cholangiocarcinoma (CCA) is a rare malignancy of the biliary epithelium, accounting for approximately 3% of gastrointestinal cancers. The clear-cell variant of CCA is rare, with only nine documented cases worldwide. This case report describes a unique presentation of clear-cell intrahepatic (or peripheral) cholangiocarcinoma (ICC), highlighting its clinical course, histopathological features, and management.

**Case Presentation:** A 56-year-old female with no significant medical history presented with postprandial right upper quadrant and epigastric pain. Following an initial diagnosis of gallbladder stones, laparoscopic cholecystectomy was performed. However, persistent epigastric pain, nausea, and vomiting led to further evaluation, revealing a 2.7 × 2.5 cm mass in the gallbladder bed compressing the common bile duct with associated intrahepatic biliary dilation. Endoscopic retrograde cholangiopancreatography confirmed the findings, and the patient underwent a left hemihepatectomy with radical choledectomy and biliary reconstruction. Histopathology demonstrated clear-cell ICC. Postoperatively, the patient received adjuvant chemotherapy and remained disease free at 14 months' follow-up.

**Methods:** A systematic review and meta-analysis were conducted according to PRISMA guidelines and the Cochrane Handbook. A comprehensive search of PubMed and SCOPUS was performed without restrictions until December 2024 to identify case reports and case series of clear-cell CCA. Data on clinical presentation, diagnostic findings, treatment modalities, and outcomes were extracted and analyzed. The quality of included studies was assessed using the Joanna Briggs Institute critical appraisal tools.

**Results:** The systematic review included 7 studies reporting 9 cases of clear-cell CCA. The mean patient age was 59.7 years, and 77.8% of the cases underwent surgical intervention. Outcomes varied, with disease-free survival ranging from 7 to 30 months in the reported cases. Factors such as tumor size and the presence of metastasis influenced prognosis.

**Conclusion:** Clear-cell CCA, though rare, should be considered in the differential diagnosis of biliary masses. Early surgical intervention is pivotal for improved outcomes, but further studies are needed to develop consensus treatment guidelines. Reporting additional cases is essential to enhance understanding and optimize management of this rare malignancy.

## 1. Case Presentation

A 56-year-old female, with no significant past medical history, presented with postprandial right upper quadrant (RUQ) and epigastric abdominal pain. Abdominal ultrasound showed multiple gallbladder stones, and hence, laparoscopic cholecystectomy was performed uneventfully. Histopathological examination of the cholecystectomy specimen revealed a well-circumscribed, pale-yellow to tan mass measuring 2.7 × 2.5 cm located at the proximal biliary tract. Microscopy demonstrated a tumor composed of nests, cords, and acinar/tubular structures lined by hyperchromatic epithelial cells with abundant clear or pale eosinophilic/vacuolated cytoplasm and round to oval nuclei showing mild to moderate pleomorphism. The tumor was associated with a desmoplastic stroma, with over 80% of cells showing clear cell change. No lymphovascular invasion was identified, and surgical margins were negative.

Over the following 2 months, the patient continued to complain of persistent epigastric pain, along with vomiting and nausea. On physical exam, she had generalized debility but no scleral icterus. A computed tomography (CT) scan was obtained, which showed a round, well-defined 2.7 × 2.5 cm mass within the gall bladder bed compressing and causing mild dilation of the common bile duct to a diameter of 1.1 cm, with moderate intrahepatic biliary dilation, worse on the left side. The liver was enlarged (to 24.5 cm in maximum dimension), with significant fat deposition within the right hepatic lobe. The overall impression was suggestive of a hilar mass causing biliary obstruction and hepatomegaly with hepatic steatosis.

**Figure 1:**
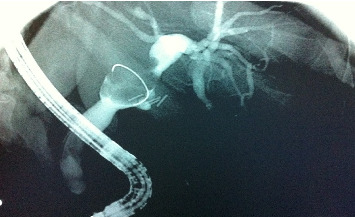
Endoscopic retrograde cholangiopancreatography (ERCP) showing intrahepatic and extrahepatic biliary dilation, and a less-visualized left intrahepatic biliary system from a stricture.

Endoscopic retrograde cholangiopancreatography (ERCP) was performed and showed a mass within the left intrahepatic biliary tree, along with dilation of the intrahepatic ducts that was more prominent on the left side, as well as mild dilation of the common bile duct (Figure 1). The patient underwent a successful left hemihepatectomy with radical choledectomy and biliary reconstruction. Postoperatively, she did well with no complications, and she was discharged home after 9 days.

**Figure 2:**
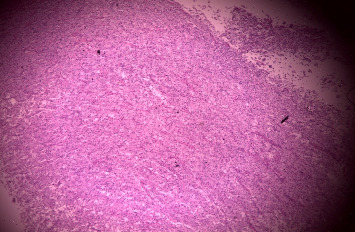
Hematoxylin and Eosin (H&E) stain low power field showing abundance of densely packed malignant cells.

**Figure 3:**
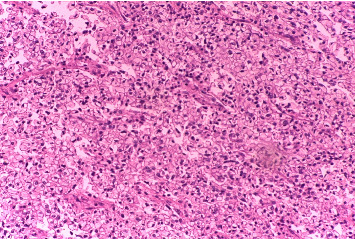
Hematoxylin and Eosin (H&E) stain showing nests and closely packed glandular structures, lined by hyperchromatic epithelial cells with clear cytoplasm. The pathological findings are consistent with a clear-cell variant of intrahepatic cholangiocarcinoma.

**Figure 4:**
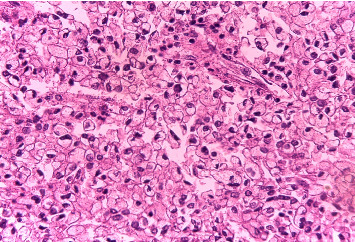
High power field of Hematoxylin and Eosin (H&E) stain showing that the majority of the cells have a pale, eosinophilic, or vacuolated cytoplasm with a clear and abundant cytoplasm resembling goblet cells. There is a distinctive overgrowth of clear cells in an acinar pattern.

Histopathology of the resected mass showed a tumor consisting of nests and closely packed glandular structures, lined by hyperchromatic epithelial cells with clear cytoplasm. The pathological findings were consistent with a clear-cell variant of intrahepatic (or peripheral) cholangiocarcinoma (ICC) (Figures 2, 3, and 4). The final pathological staging was T1N0M0, corresponding to Stage I disease, indicating a solitary tumor without vascular invasion.

Postoperatively, the patient received adjuvant chemotherapy with gemcitabine and cisplatin, and follow-up after 14 months showed no evidence of recurrence, and the patient was doing well.

## 2. Introduction

Cholangiocarcinoma (CCA) is a cancer originating from the biliary tract epithelium, comprising about 3% of all gastrointestinal malignancies. Autopsy-based studies have reported its occurrence in approximately 0.01%–0.46% of the cases, underscoring its uncommon nature. Although adenocarcinoma is the predominant histological type, accounting for over 90% of the CCA cases, rare subtypes such as mucinous, squamous, and clear-cell carcinomas have also been identified. Clear-cell carcinoma, in particular, is extremely rare, with only nine cases documented globally [1, 2].

Clear-cell CCA is distinguished by tumor cells that exhibit a clear cytoplasm, a characteristic resulting from the loss of glycogen, phospholipids, and neutral lipids during routine histological preparation. This rare subtype generally presents in individuals in their fifth or sixth decade of life and shows no significant gender bias or known links to predisposing conditions like hepatitis. Compared with other ICC types, the clear cell variant may offer a more favorable prognosis, though its clinical behavior is still not fully understood [3, 4].

From a histopathological perspective, clear-cell CCA features cells with distinct borders and a clear cytoplasm, embedded within a richly vascularized stroma. The differential diagnosis must consider other clear cell tumors that can arise from organs such as the kidneys, pancreas, ovaries, and lower urinary tract. A precise diagnosis requires detailed histological examination combined with immunohistochemical analysis to rule out these potential mimickers [3–5].

This report describes an exceptionally rare instance of clear-cell CCA, offering comprehensive clinical and pathological observations. It also incorporates a review of the scarce available literature to improve awareness and understanding of this uncommon variant, thereby adding to the existing knowledge of CCA subtypes.

## 3. Methods

### 3.1. Protocol Registration

This systematic review and meta-analysis adhered to the Preferred Reporting Items for Systematic Reviews and Meta-Analyses (PRISMA) [6] guidelines and the Cochrane Handbook for Systematic Reviews of Interventions. The study protocol was registered in the International Prospective Register of Systematic Reviews (PROSPERO) [7] under the registration number: CRD420251006967.

### 3.2. Data Sources and Search Strategy

PubMed and SCOPUS were systematically searched by M.M.A. and G.A. up to December 24, 2024, without any search restrictions. The search strategy included terms related to clear-cell CCA and case-based study designs. The exact search terms are detailed in Table 1.

### 3.3. Eligibility Criteria

The inclusion criteria focused on studies involving patients diagnosed with clear-cell CCA, including its presentation, diagnosis, or treatment. No comparator group was required. The outcomes considered included clinical presentation, diagnostic findings, treatment modalities, and prognosis. Eligible study designs included case reports and case series. Studies were excluded if they were duplicates or written in languages other than English.

### 3.4. Study Selection

Two reviewers, M.M.A. and G.A., independently screened the titles and abstracts of all retrieved records using Rayyan online software. Full-text screening was conducted for studies that met the initial criteria, with eligibility determined based on the predefined criteria. Any disagreements between reviewers were resolved through discussion.

### 3.5. Data Extraction

Data were extracted using a pretested form developed by M.M.A. and G.A. Variables collected included patient demographics, tumor characteristics, treatment modalities, and outcomes. The reviewers performed data extraction independently, and disagreements were resolved through discussion to ensure accuracy.

### 3.6. Risk of Bias and Certainty of Evidence

The quality of included studies and their risk of bias were assessed independently by two investigators using the Joanna Briggs Institute (JBI) critical appraisal tools for case reports and case series [8]. Discrepancies in assessments were resolved by a third reviewer. Studies were classified as having a high, moderate, or low risk of bias based on the proportion of items rated as “yes.”

### 3.7. Statistical Analysis

Continuous variables were expressed as the mean ± standard deviation (SD) or median (interquartile range), based on the normality of their distribution. Categorical variables were reported as counts (percentages). All statistical analyses were performed using R software (Version 3.4.0, R Foundation for Statistical Computing, Vienna, Austria) [9].

## 4. Results

### 4.1. Search Results and Study Selection

**Figure 5:**
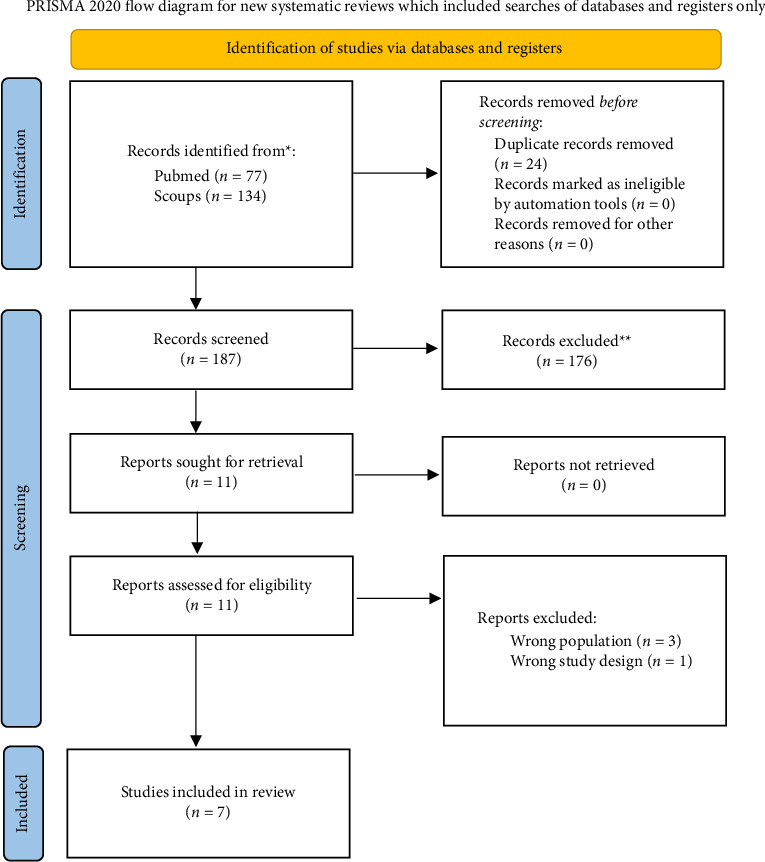
PRISMA flowchart for the included studies.

Our search yielded 211 records across the two databases, with 187 records remaining after the removal of 24 duplicates. After screening titles and abstracts, 176 records were excluded, leaving 11 studies retrieved for full-text review. Of these, 4 studies were excluded (3 due to the wrong population and 1 due to the wrong study design), resulting in 7 studies included in the final review. The detailed search and selection process is depicted in Figure 5.

### 4.2. Characteristics of Included Studies and Patients

The review included 7 studies describing 9 patients with clear-cell CCA. One study (Haas et al., 2007) detailed 3 patients, while the remaining studies each focused on a single case. The mean age of the included patients was 59.7 years (SD: 6.9), and the mean tumor size was 6.4 cm (SD: 4.7). Of the 9 patients, 6 (66.7%) were male and 3 (33.3%) were female. Metastasis was reported as “Yes” in 1 (11.1%) case, “No” or “Negative” in 3 (33.3%) cases, and was unknown in 4 (44.4%) cases.

Surgical intervention was performed in 7 (77.8%) cases, with two additional cases specifying right lobe surgery. Chemotherapy was administered in 3 (33.3%) cases, while 3 (33.3%) did not receive it, and chemotherapy details were unknown or not applicable in the remaining 3 cases.

Prognosis varied across patients: one patient achieved survival of 7 months free of recurrence, one died after 14 months without treatment, and one died after 3 years. Four patients were reported alive at different follow-up periods (12 months, 30 months, and 1 year), with one achieving a short-term survival of 7 months. Full details are presented in Table 2.

### 4.3. Risk of Bias and Certainty of Evidence

The risk of bias assessment revealed variability among the included studies. Two studies, Albores-Saavedra J 2001 and Haas S 2007, were categorized as having a low risk of bias due to comprehensive and clear reporting across most domains, including patient demographics, diagnostics, and treatment. Three studies, Adamek HE 1998, Falta EM 1999, and Toriyama E 2010, were classified as having a moderate risk of bias, primarily due to incomplete reporting of diagnostic methods, follow-up outcomes, or adverse events. Lastly, two studies, Logani S 1998 and Tihan T 1998, were identified as having a high risk of bias, reflecting significant gaps in reporting on clinical conditions, diagnostic tests, treatment details, and postintervention outcomes. These findings highlight inconsistencies in the methodological quality and reporting standards among the included case reports.

## 5. Discussion

CCA, a rare gastrointestinal tumor, continues to present challenges in diagnosis and treatment. Newer radiologic techniques, including dynamic CT, magnetic resonance imaging (MRI), magnetic resonance cholangiopancreatography (MRCP), and positron-emission tomography (PET) have been allowing a more reliable preoperative staging [3]. In patients who are considered potentially resectable, careful preoperative planning should be carried out to increase the possibility of achieving a histologic margin-negative resection, as this improves longer-term survival [1–3].

In the late 1990s, a new variant of CCA, clear-cell carcinoma, was described. Nine cases have been reported in the literature since then (Table 2). As the number of cases is very low, there is not a clear definition of histology and no clear association of risk factors. This type generally consists of a glandular and trabecular growth pattern with abundant desmoplastic stroma and clear cell changes in about 80% of the tumor cells. Histopathologic review shows mostly intrahepatic tumor distribution [9–11].

ICC arises from any portion of the intrahepatic bile duct epithelium, whether large or small bile ductal branches. On the other hand, CCA arising from the right or left hepatic ducts or at the bifurcation defines the hilar CCA subtype, which is considered an extrahepatic lesion [1]. Most of the ICC show tubular and/or papillary structures with a variable fibrous stroma. There is no dominant histologic type of ICC in cases associated with liver flukes or hepatolithiasis when compared with those in nonendemic areas [3, 4].

Adenocarcinoma type of ICC growing into the hepatic parenchyma and portal pedicle reveals a significant heterogeneity of histologic features and degree of differentiation. At an early stage, a tubular pattern with a relatively uniform histologic picture is frequent. Cord-like or micropapillary patterns are also seen. The cells could be small or large, cuboidal or columnar, and can be pleomorphic. The nucleus is small and the nucleolus is usually less prominent than that of hepatocellular carcinoma (HCC) [1–4].

In the rare variant of clear-cell carcinoma, the majority of the cells have a pale, eosinophilic, or vacuolated cytoplasm; sometimes, the cells have a clear and abundant cytoplasm or resemble goblet cells. Clear-cell variant is characterized by distinct overgrowth of clear cells in an acinar or tubular pattern. The tumor cells are periodic acid-Schiff (PAS) reactive and diastase resistant, indicating the presence of mucin [3, 4].

Among the published cases of the clear cell variant of cholangiocarcinoma (CCC), outcomes and treatments vary significantly, reflecting the rarity and complexity of this disease. One notable case by Adamek et al. [11] involved a patient with hepatitis B virus (HBV) infection who received antiviral therapy with lamivudine, later switched to entecavir, achieving seroconversion. This case highlights the importance of managing underlying liver conditions to potentially improve outcomes.

Chemotherapy was administered postoperatively in two cases. Toriyama [1] reported a patient treated with Tegafur–Gimeracil–Oteracil potassium (S-1), achieving 7 months of disease-free survival. In another instance, Haas et al. [4] described a patient who received postoperative chemotherapy but died 3 years later following recurrence and metastasis. These examples suggest that while adjuvant chemotherapy may be beneficial in prolonging survival, its efficacy remains inconclusive given the limited data.

Surgical resection remains the cornerstone of treatment for this rare variant. For example, Logani and Adsay [10] reported a 64-year-old female who underwent surgery and achieved 7 months of disease-free survival, although long-term follow-up was not available. Similarly, Tihan et al. [12] documented a 72-year-old male who survived 30 months after resection. Albores-Saavedra et al. [5] reported a 64-year-old male who remained alive for 3 years postoperatively. The present case adds to this evidence, with no recurrence observed at 14 months following surgery, underscoring the importance of early surgical intervention.

Tumor size and metastasis appear to play significant roles in outcomes. For example, Tihan et al. [12] described a patient with a large 15-cm tumor who underwent resection and achieved long-term survival without metastasis. In contrast, Haas et al. [4] reported a patient with a 9-cm tumor and metastasis who died 3 years after surgery. Smaller tumors, such as the 2.7-cm tumor in the present case and the 2.2-cm tumor in the case by Toriyama [1], were associated with better outcomes and longer recurrence-free periods.

Exceptions to the generally favorable prognosis of clear-cell CCC include cases where surgery was not feasible. For instance, Adamek et al. [11] reported a patient who could not undergo resection due to underlying diseases and died 14 months after diagnosis without treatment. This highlights the critical role of patient selection and the potential impact of comorbidities on prognosis.

Our case of intrahepatic clear-cell CCA was unique from the other reported cases in its bulky nature, with no local spread or distant metastasis, and interestingly, the mass did not exert a significant mass effect to result in a tight stricture or jaundice, supporting a better prognosis. We explain that the patient presented relatively early in the course of the disease after she had cholecystectomy.

Currently, there are no consensus guidelines for the treatment of clear cell CCC due to its rarity. Treatment approaches are largely extrapolated from those used for other CCA variants. The need for neoadjuvant or definitive postoperative chemotherapy cannot yet be determined due to the limited number of cases. However, careful follow-up and individualized consideration of adjuvant therapy appear essential for optimizing outcomes.

In conclusion, while most reported cases of clear-cell CCC demonstrate relatively favorable outcomes, this variant requires further investigation to establish evidence-based management strategies. Collaborative efforts and documentation of additional cases are crucial to understanding the role of chemotherapy, the impact of tumor characteristics, and the long-term benefits of surgical intervention for this rare malignancy.

## Figures and Tables

**Table 1:** Description of the search strategy used in each database.

Database	Search terms (search date: 24/12/2024)	Search field	Search results
PubMed	(“Clear-cell cholangiocarcinoma” OR “clear cell cholangiocarcinoma” OR “clear-cell carcinoma of the bile ducts” OR “clear cell bile duct carcinoma” OR “clear cell carcinoma of the biliary tract”) AND (“case report” OR “case reports” OR “case series” OR “case study” OR “case studies” OR “clinical report”)	All field	77
SCOPUS	Clear cell cholangiocarcinoma AND (“case report” OR “case series”) article type filter: Case reports	Title, abstract, keywords	134

**Table 2:** Case reports of clear-cell cholangiocarcinoma.

	Age (years)	Gender	Mass size (cm)	Mets	Surgery	Location	Chemotherapy	Prognosis
Logani and Adsay [10]	64	Female	12	Unknown	Yes	Unknown	Yes	7 moths free survival, unknown later
Adamek et al. [11]	62	Male	5	Negative	No	Right lobe		Died after 14 months, without treatment
Tihan et al. [12]	72	Male	15	Negative	Yes	Left lobe		Alive at 30 months
Falta et al. [2]	50	Male	1.5	Negative	Yes	Segment VI	NA	Alive at 12 months
Albores-Saavedra et al. [5]	64	Male	6	Negative	Yes	Right lobe		Alive 3 years
Haas et al. [4]	51	Male	9	Yes	Yes. Recurrence 1 year later, s/p liver transplant	Segment IVa. 1 year later had segments III, V involved	Yes	Died after 3 years
Haas et al. [4]	60	Female	4.5	Unknown	Yes	Segment V	No	Alive 1 year
Haas et al. [4]	58	Female	2.3	Unknown	Yes	Segment V	No	Alive
Toriyama [1]	56	Male	2.2	Negative	Yes	Segment VII	Yes	Alive 7 months
Present case	56	Female	2.7	Negative	Yes			Follow-up of 14 months showed no evidence of recurrence, and the patient was doing well.

## Data Availability

Data sharing not applicable to this article as no datasets were generated or analyzed during the current study.
